# Analyze the binding of amyloid beta 1‐42 to specific regions of the TREM2 gene

**DOI:** 10.1002/alz70855_106314

**Published:** 2025-12-24

**Authors:** Seçil Dülger, Zuhal Yurttaş, Tugay Çamoğlu, Erdinc Dursun, Duygu Gezen‐Ak

**Affiliations:** ^1^ Institute of Neurological Sciences, Istanbul University‐Cerrahpaşa, Istanbul, Turkey

## Abstract

**Background:**

Alzheimer's disease (AD) is the most prevalent neurodegenerative disorder. The amyloid beta (Aβ) peptide plays a crucial role in the pathophysiology of AD. Similar to transcription factors, studies have shown that Aβ can translocate to the nucleus, where it binds to or functionally interacts with specific DNA sequences. Aβ interacts with the 'KGGRKTGGGG' sequence in the promoters of the *APOE, APP*, and *BACE1* genes, potentially acting as a transcription factor for these genes. Additionally, it has been demonstrated that Aβ1‐42 alters the expression of the *TREM2* gene. Furthermore, Aβ is known to be one of the ligands for the TREM2 receptor. In this study, we investigated the binding potential of Aβ to the regulatory sequences of the *TREM2* gene.

**Method:**

Sequences showing homology with the DNA‐binding consensus sequence of Aβ were searched in the upstream, downstream, and non‐coding regions of the *TREM2* gene. Four regions with homology were selected. HEK293T cells were treated with 1 μM Aβ1‐42 peptide. After 24 hours, chromatin immunoprecipitation (ChIP) was performed using Aβ‐specific antibodies. Quantitative polymerase chain reaction (qPCR) was performed on the obtained DNA fragments. The qPCR‐ChIP results were calculated using the fold enrichment method.

**Result:**

We showed that Aβ interacted with the regulatory DNA regions of the *TREM2* gene, including the enhancer located upstream, intron 2, and non‐coding exon 5. The signal from the enhancer region was higher than that from the others. (Fold enrichment values above 3 were considered binding signals.)

**Conclusion:**

Our results showed that Aβ interacts with the regulatory DNA regions of the *TREM2* gene. The data obtained will contribute to the identification of a new mechanism, beyond the receptor‐ligand relationship, involving Aβ and TREM2 in the pathological mechanisms of AD, and to the understanding of complex processes related to AD. Our study is also expected to provide insights into TREM2‐related therapeutic approaches for AD. This study is supported by the Health Institutes of Türkiye (TUSEB) (Project ID: 37319).

